# Social enterprise as a strategy to advance patient-oriented health services innovation: learning from the Alberta Family Integrated Care model

**DOI:** 10.3389/frhs.2025.1638587

**Published:** 2025-10-02

**Authors:** Anmol Shahid, Kristen Graham, Karen Benzies

**Affiliations:** 1 Faculty of Nursing, University of Calgary, Calgary, AB, Canada; 2 Faculty of Nursing, Alberta Health Services, Calgary, AB, Canada; 3 Faculty of Nursing, Liminality Innovations, Calgary, AB, Canada

**Keywords:** delivery of care, neonatal, intensive care units, learning health system, sustainability, social innovation, social enterprise, critical care

## Abstract

This community case study outlines the conceptualization, development, implementation, and commercialization of the Alberta Family Integrated Care (Alberta FICare) model, offering insights into a unique way of sustaining patient-oriented innovations through social enterprise. Our team developed the Alberta FICare model to include families as partners in care in neonatal intensive care units (NICUs). Research phases of our model showed improved outcomes for neonates (shorter hospital stays), their families (greater caregiving self-efficacy, reduced psychosocial distress), and the health system (cost avoidance). Despite co-development of the model with families, providers, and leaders, rigorous testing (cluster randomized controlled trial), and province-wide scale-up (now standard of care in all 14 Alberta NICUs) efforts to sustain the model stalled due to shifting health system priorities. To address this challenge, we incorporated a social enterprise *(Liminality Innovations Inc.)* to sustain the model of care and support broader dissemination of family integrated care practices in NICUs beyond Alberta. While this strategy fostered sustainment and growth of our model, it also raised challenges. Some of these challenges included tackling perceptions within the research and practice communities that commercialization undermines research integrity. We share our experiences to highlight the potential of ethical, mission-driven commercialization through social enterprise to support innovation in learning health systems through ongoing interest holder engagement, responsible stewardship, and improving learning health system outcomes as the central goal.

## 1 Introduction

### 1.1 Needs of families in neonatal intensive care units

Globally, about one in ten infants is born preterm, and many need specialized support in neonatal intensive care units (NICUs) (1). Amidst goals to reduce mortality and morbidity, healthcare providers in NICUs may unintentionally limit parental involvement in care and create feelings of marginalization for parents of neonates (2). This oversight can heighten parental stress, anxiety and depression, disrupt the parent-infant relationship, and reduce parental confidence in ability to care for their child long-term (3–5). Parental demand for greater involvement in the care of their critically ill newborn and recognition by healthcare providers that parental care improved infant outcomes, has challenged traditional models of NICU care (6).

To address the limitations of traditional models of care in NICUs, our team led by Dr. Karen Benzies, developed the Alberta Family Integrated Care (Alberta FICare) model. This model was designed to empower families to become integral members of the NICU care team, fostering a more supportive environment for parents of critically ill neonates. This model is centered around creating educational modules for healthcare providers working in NICU settings and creating a scaffolding of support from our team, unit and system-wide leadership, and site champions to roll out this education to NICUs. Core elements of the model are described in Section 2.1 and corresponding subsections. Facing challenges in sustaining the model in local NICUs after a successful rollout into practice, our team took a social enterprise approach to enable sustainment and ongoing scale and spread of Alberta FICare.

### 1.2 Using the community case study to reflect on innovation within learning health systems

We detail and reflect on our journey with the Alberta FICare model using case study methodology to highlight the contrast between the observed and theoretically predicted events in our model's research to practice trajectory (7, 8). This methodology has served as both informal and formal components of intervention evaluation in health services, using reflection exercises to contribute to the overall evaluation process (7). A modified case study approach (i.e., community case study) is used here to reflect on the operation of our model in a learning health system (LHS), where “fuels and accelerants” (e.g., leadership, key partnerships with patients, community and leadership, engagement with equity-deserving groups and the health workforce, implementation and decision supports, learning networks) or “moderators/breaks” (i.e., health system capacity, governance), as described in the Learning Health System Action Framework can influence outcomes generated by an intervention (9).

Thus, for the purposes of this work, we define a community case study in accordance with the Smith et al. definition (2016) as “a description of, and reflection upon, a program or practice geared toward improving the health and functioning of a targeted population” (10). We acknowledge this methodology highlights “community” in contrast to “clinical” studies, while recognizing that “community” may be defined by geographic boundaries, demographic characteristics, common settings, and/or affiliations (9). Looking into broader guidance around community-style case studies, this methodology also represents activities across different research stages (i.e., early development of an innovation to later phases), such as documenting the implementation of an evidence-based program in a new context (e.g., different culture, population, or setting) than the one in which it was originally developed as part of the “from science to practice” trajectory (11). It is notable that the “case” for the value of case studies is championed by those who believe that observation and reflection constitute empirical inquiry capable of investigating a contemporary phenomenon (the “case”) in depth and within its real-world context (12).

## 2 Context

### 2.1 Alberta FICare: a model to standardize family integration in NICUs

Alberta FICare was developed as a direct and deliberate response to the need to transform NICU practices by including families as partners in care. The creation of this model was supported by evidence of the benefits of family-centered care across hospitalized patient populations (13–18). While there has been a shift towards family engaged care in pediatric populations, many efforts have been targeted at one group of individuals (i.e., patients, mothers, or staff) or set of outcomes (19). The Alberta FICare approach marks a shift from single component to a multi-component intervention with a more holistic, standardized approach that positions families as essential members of the care team and also improve outcomes across important to LHSs (patient experience, provider experience, population outcomes, and value for the health system)^1^ (20). At its core, the model affirms the vital role of parents, recognizing the unique insights, knowledge, and relationship they bring to their infant's journey toward recovery.

#### 2.1.1 Elements and enablers of the Alberta FICare model

Moving beyond traditional models that focus on the infant's medical needs in relative isolation, Alberta FICare promotes a holistic approach to NICU care through tools and strategies centered on core elements of (1) education, (2) relational communication, and (3) support. These core elements are designed to function synergistically with key enablers (i.e., creating a family friendly NICU space, partnerships) to support a culture shift in NICUs to truly foster family-integrated care.

##### 2.1.1.1 Education (core element)

A cornerstone of Alberta FICare is its educational modules, designed for staff including healthcare professionals (physicians, nurses, allied health) and unit clerks. These asynchronous online modules are grounded in Adult Learning Theory principles of self-direction, readiness, relevance, and impact (21). Developed and refined in collaboration with key interest holders, the modules are responsive to the needs of both NICU staff and parents from diverse backgrounds. They include staff education centered on equity including involving partners or non-birthing parents in care, and being mindful of the needs of single parents, same-sex parents, individuals with differing linguistic and cultural backgrounds, newcomers, and others.

##### 2.1.1.2 Relational communication (core element)

Alberta FICare unit staff training emphasizes relational communication strategies to integrate parents of NICU patients into the care team. The training encourages reflections on past interactions with parents of NICU patients and considers how changes in communication methods could have influenced these interactions. The training also promotes using question styles that empower both staff and families to more effectively share their experiences and insights (22, 23). Participants practice using these questions and reflect on their application in clinical settings. Finally, Alberta FICare urges staff to recognize consistent, positive parental behaviors as a strategy to build trust (22).Together, the relational communication strategies of Alberta FICare equip NICU staff to navigate a wide range of conversations with parents of hospitalized neonates.

##### 2.1.1.3 Support (core element)

Alberta FICare was designed with a deep understanding of the emotional and practical challenges families face when their infant is admitted to the NICU from birth. Ongoing consideration of factors important to families (e.g., out-of-pocket expenses associated with hospitalization) are noted in the application of the model (24). In addition to traditional support from social workers, Alberta FICare encourages the adoption of peer family mentors who can listen and share their experiences.

##### 2.1.1.4 Role redefinition in NICUs (enabler)

Alberta FICare redefines the role of parents in the NICU by actively encouraging and supporting their participation in all aspects of their infant's care as soon as they are ready and willing after admission. Parents are encouraged to engage in routine caregiving tasks (e.g., feeding, diapering, bathing, and providing comforting touch), as appropriate. These roles foster vital parent-infant relationships, enhance parental confidence, and support healthy infant development (25, 26). Parents are also invited to take part in bedside medical rounds and care planning meetings, where they are encouraged to introduce themselves, share observations, ask questions, and contribute meaningfully to decisions about their infant's individualized care plan. With evidence-based roots, this collaborative approach ensures care is aligned with the unique needs, preferences, and values of both the infant and family (27). By integrating parents of NICU patients in care teams, Alberta FICare empowers them to serve as informed and confident advocates for their neonate's well-being.

##### 2.1.1.5 Creating a family friendly NICU space (enabler)

Alberta FICare's elements are supported by suggestions to create a family friendly space from the Mount Sinai FICare model recognizing that space impacts the ability of families to participate meaningfully in care.^2^ NICUs have adapted their physical spaces to better accommodate families, including welcoming signage, sleeping options for parents, family lounges with microwaves, fridges, and reclining chairs to enable parents to rest and provide skin-to-skin care. We consider these environmental modifications enablers of the success of Alberta FICare.

##### 2.1.1.6 Partnerships (enabler)

The elements and enablers of Alberta FICare are implemented in partnership with all levels of interest holders within LHSs (e.g., patients, families, healthcare providers, unit staff, and health system leaders). Alberta FICare demonstrates how meaningful partnerships can act as powerful enablers of innovation and drive improvements in outcomes that matter to LHSs. Local collaborations (particularly with the former Maternal, Newborn, Child and Youth, Strategic Clinical Network) were central to the success of the model. These partnerships aided efforts to secure provincial grant funding for research, gain broader health system support, and enable the spread and scale of Alberta FICare (28). Leaders and administrators within our local health system played a pivotal role by advocating for policy changes, securing necessary resources, and facilitating implementation across diverse clinical settings.

### 2.2 Development to implementation

The development to implementation process of Alberta FICare followed Waye's Innovation Pipeline, which has supported our local health system to continue to improve the delivery to service through innovation through a series of sequenced steps (i.e., idea generation, pilot test, test of implementation, test of implementation while spreading, implement to sustain care) (29). Alberta FICare was co-developed through a collaborative process that brought together a multidisciplinary team of interest holders, each contributing essential expertise and perspectives. Our team (including researchers from the University of Calgary who led the design and evaluation processes), drew on their expertise in methodology, evidence synthesis, and health services research. Healthcare providers (including nurses, physicians, and allied health professionals) were asked to share insights from the frontlines of NICU care, while families with lived experience were consulted to ensure that the model addressed their needs.

The testing and implementation of Alberta FICare followed a carefully structured and phased approach, designed to ensure its seamless integration into routine clinical practice. Initial pilot studies were strategically conducted in select NICUs within Alberta to rigorously assess the feasibility and acceptability of the model, allowing for valuable feedback and iterative refinement of its components. Building upon the insights gained from the pilot phase, Alberta FICare underwent rigorous evaluation through a cluster randomized controlled trial, showing a significant reduction in infant hospital length of stay (16). There were trends favoring Alberta FICare in reduced maternal psychosocial distress and increased self-efficacy (30). With reduced infant length of stay, the health system avoided costs (31). With robust evidence supporting Alberta FICare and the support of our collaborators, Alberta FICare was scaled to all 14 NICUs across Alberta, helping to sustain a province-wide shift toward family-integrated NICU care.

## 3 The challenge of sustaining Alberta FICare, and a solution

The rollout of Alberta FICare in NICUs across the province marked a significant achievement in translating research evidence into practice and demonstrated our local LHS's commitment to evidence-based care and patient-centered innovation. This province-wide implementation reflected the collaborative efforts of all interest holders and their shared dedication to improving care for infants and families in Alberta. The context of our local LHS, characterized by its emphasis on evidence-based practice, created a supportive environment for the development, implementation, and scaling of Alberta FICare. However, the inherent complexities of a large health system, including competing priorities, resource constraints, and the need for iterative revisions in response to evolving circumstances, also presented challenges that required careful navigation and proactive strategies to ensure the long-term sustainability of the model.

### 3.1 The challenge

Alberta FICare showed promise in its development (co-development with active participation from families, healthcare providers, and healthcare leaders) and its rigorous testing phases. However, we found the long-term use of Alberta FICare at sufficient intensity for the sustained achievement of desirable program goals and population outcomes (i.e., sustainability defined by Scheirer & Dearing) (32) of the model within our local health system to be challenging. Shifting local health system priorities led to a stagnation of ongoing efforts to sustain the model when positions assigned to sustain this project within the system were terminated due to restructuring. This stalling of momentum raised critical questions about the continued impact and long-term viability of Alberta FICare, highlighting a pervasive and persistent challenge within healthcare innovation (33). Promising patient-oriented research innovations frequently face the risk of being deprioritized or even abandoned in their ongoing integration into LHSs, primarily due to the limitations of traditional funding models and the ever-present pressures on healthcare budgets (34). Traditional research funding models, while essential for initial discovery and early-stage development and implementation, often fall short in providing adequate support for later phases of innovation, including the complex and resource-intensive processes of long-term sustainment (35, 36). This funding gap creates a significant vulnerability, threatening to undermine the potential for widespread and lasting impact of valuable healthcare advancements (32).

### 3.2 The solution: embracing a social enterprise approach

Amidst the sustainability challenges we faced, which appears to be common to multi-component interventions in complex health systems (33), we made a deliberate decision to explore commercial strategies to sustain Alberta FICare. This led to the founding of *Liminality Innovations Inc.*, (Founder and CEO: Karen Benzies) a social enterprise dedicated to supporting the ongoing research, development, and dissemination of Alberta FICare (rebranded as Merge™).^3^ Social enterprises operate within the Social and Solidarity Economy framework, a paradigm that prioritizes social objectives alongside economic viability. Rather than distributing profits to shareholders, revenue generated by social enterprises is reinvested into achieving specific social missions, such as improving healthcare delivery or enhancing community well-being. Social enterprises frequently adopt equitable labour practices, empower workers with greater control over their work, and contribute to psychological well-being and social cohesion (37). By aligning with this framework, *Liminality Innovations Inc.* aimed to support the sustainment of Alberta FICare and enhance its potential to address broader social determinants of health.

*Liminality Innovations Inc.* was also suited to tackle sustainability of Alberta FICare due to social enterprises being well-suited to LHS innovations because they enable nimble, values-aligned responses to community needs. They can deliver flexible programming, adapt materials quickly to diverse contexts, and foster a sense of ownership and connection among users (qualities often lacking in traditional institutional models). Moreover, social enterprises contribute to community capacity by building individual and collective assets, such as health and evaluation knowledge, communication skills, and supportive peer networks (37–39).

### 3.3 Lessons learned and practical implications for healthcare innovation

Our team's experience with the Alberta FICare model has yielded invaluable lessons, offering insights we are now able to share with others. We hope these insights will support other patient-oriented health services innovators, particularly those navigating the evolving landscape of LHSs.

We recognize several strengths in our journey. Our work was rooted in co-design (i.e., deliberately involving interest holders at every stage), and guided by the phased Innovation Pipeline approach to innovation in LHSs (29). We employed rigorous research methodology, including a randomized controlled trial, which provided compelling evidence of Alberta FICare's effectiveness. We also prioritized evaluating the model's economic value, both at the industry level and in terms of out-of-pocket costs for parents (24, 40). Demonstrating this economic impact was key to securing buy-in from healthcare leaders and enabled the successful scaling of the model across Alberta.

However, the commercialization of Alberta FICare through *Liminality Innovations Inc.* introduced challenges, prompting our team to develop a mitigation strategy for future efforts. Many of these challenges arose from perceptions that commercialization (including social enterprise for those less familiar with the concept) is inherently profit-driven rather than focused on societal benefit. This perception is common in health services research, where commercialization can be met with skepticism due to concerns that profit motives might undermine ethical care delivery or academic integrity (41).

In our experience, addressing these perceptions required deliberate strategies such as partnership-building, responsible stewardship of the social enterprise, and clearly demonstrating the need to address sustainment challenges. These strategies, combined with an unwavering commitment to the core mission of Alberta FICare to elevate family-centered care proved valuable in mitigating concerns around commercialization. The strategies we employed, along with our reflections on their use, are described below.

#### 3.3.1 Creating lasting and trusting partnerships

We found that a helpful strategy for mitigating skepticism around commercialization in research is the development of lasting and trusting partnerships. Our experience with Alberta FICare underscored the critical role those strong collaborations (particularly with interest holders within our local health system) play in legitimizing and supporting a commercialization approach. Our partners recognized that commercialization through social enterprise could offer a practical mechanism for sustaining the model amid shifting health system priorities by generating cost-recovery revenue through expansion to other jurisdictions. This revenue, in turn, could help fund and preserve the model locally. Our co-design approach was foundational to building these partnerships, as it ensured the model was relevant, acceptable, and feasible across settings. More importantly, it cultivated a deep sense of ownership and shared responsibility among partners, which proved essential not only for successful implementation but also for the long-term sustainability of Alberta FICare.

#### 3.3.2 Responsible stewardship

To address the perception that commercialization may conflict with research integrity, we prioritized responsible stewardship of the Alberta FICare social enterprise by maintaining a clear and consistent focus on our core goal: improving care for patients, families, and the health system. From the outset, we communicated this unwavering commitment to all collaborators and peers, signaling that financial viability would not come at the expense of patient-centered values or scientific rigor. Transparency played a central role in this approach, and we were not hesitant to share the information that earnings generated through the social enterprise were reinvested to support and sustain the model. This transparency, combined with a strong ethical lens and ongoing oversight, reinforced our dedication to ethical practice. In our view, commercialization by social enterprise should serve as a vehicle to enhance, rather than detract from, the values at the heart of health services innovation.

#### 3.3.3 Highlighting the capacity of social enterprises to fill a gap in sustainment

We found that highlighting the capacity of social enterprise to sustain models beyond traditional funding cycles is key to addressing misconceptions about its role in health services research and LHSs. Our experience with Alberta FICare underscores a well-known limitation of research funding: while it often supports the development and early implementation of innovations, it rarely extends to their long-term sustainment or adaptation within evolving health systems. LHSs, despite their strengths in translating evidence into practice, are not immune to shifting priorities and resource constraints. This makes it essential for researchers and decision-makers to develop a deeper understanding of what sustainment of an effective intervention truly entails. Rather than simply maintaining an intervention over time, ensuring its relevance, adaptability, and integration into routine care is important. In this context, the creation of a social enterprise *(Liminality Innovations Inc.)* offered a viable and ethically grounded pathway for sustaining Alberta FICare beyond initial funding windows. By generating revenue through wider (beyond Alberta) dissemination of the model, we were able to support ongoing maintenance, such as updating educational modules, and foster further innovation. This approach demonstrated that commercialization, when aligned with patient-oriented goals, can complement rather than compete with the mission of improving care. In doing so, it helped to reframe social enterprise not as a threat to research integrity, but as a valuable and responsible mechanism for long-term impact.

#### 3.3.4 Metrics and early impacts of our social enterprise

*Liminality Innovations Inc.* is a young organization and has published research papers on Alberta FICare metrics such as health system utilization and cost avoidance for the health system and out of pocket costs for parents (24, 30, 31, 40). However, early indicators for these metrics suggest strong potential for our company's growth and impact. However, we can report the governance structure is comprised of a mix of academic researchers and business executives. Finally, any revenue generated by *Liminality Innovations Inc.* is reinvested into operational costs and further research and development of Alberta FICare. Pricing of the model so far has been based on a cost-recovery model and supplemented by partnership grant ensuring accessibility for diverse health systems. Furthermore, we have successfully expanded the Alberta FICare model to several national (other provinces in Canada) and international NICUs (East Asia), demonstrating its potential for scalability and reach. As the company grows, we anticipate sharing more about our social enterprise's commitment to equitable pricing, revenue and reinvestment figures.

#### 3.3.5 *Liminality innovations Inc.* in relation to other social enterprises in health

*Liminality Innovations Inc.* occupies a unique position within the global landscape of healthcare-related social enterprises (42), sharing limited common ground with models such as Grameen Healthcare in Bangladesh (financed by a local bank) (43), Programa Saúde da Família in Brazil (44), and Aravind Eye Care in India (45). Although *Liminality Innovations Inc.'s* Alberta FICare is primarily an educational product (i.e., distinct from the hospitals and clinics often highlighted in the social innovation literature) it maintains a socially driven mandate while leveraging cost-recovery pricing and reinvestment strategies to support ongoing innovation and maintains its commitment to equity and sustainability. The growth of *Liminality Innovations Inc.* in the coming years is expected to yield insights into the model's actual impact, including revenue generation, value delivered to health systems and their users, and potential applicability in settings beyond NICUs.

## 4 Acknowledgment of conceptual constraints and conclusion

This community case study is grounded in the experiences of our team and may not be directly generalizable to all contexts or types of health service innovations, even within the realm of social enterprise. While the social enterprise approach has shown promise for the sustainment and growth of Alberta FICare, it remains a relatively new strategy in health services research within LHSs, and its long-term benefits warrant further investigation.

Methodologically, this case study is descriptive, offering practical insights into the challenges and opportunities encountered during implementation. As such, it does not include the controlled comparisons typical of traditional research designs and therefore cannot definitively attribute observed outcomes to the social enterprise model alone. One of the main criticisms of case study research is that it is interpretive and inherently open to subjective assessment. However, we use the community case study format to illustrate a learning opportunity for researchers addressing sustainability challenges of their innovations in LHSs and find the methodology appropriate for knowledge sharing in this context (7, 10). Future research using more rigorous evaluation methods could help assess the effectiveness of commercialization strategies in sustaining and spreading other health service innovations.

To conclude, this community case study highlights the need for open dialogue and increased awareness within the health services research community about the ethical and practical implications of commercialization by social innovation, as well as its potential to support the sustainability of innovations within LHSs. Looking ahead, researchers and healthcare leaders could benefit from exploring sustainability strategies including the potential role of social enterprise early in the innovation lifecycle with partnerships, responsible stewardship, and the value of social enterprise in sustaining innovations in mind. Finally, longitudinal evaluation of social enterprise models is essential to understanding the value that organizations such as *Liminality Innovations Inc*. can offer LHSs in sustaining complex health system interventions.

### Glossary (terms used in this manuscript in the context of health services)

- Commercialization—Turning a research-based health intervention, product, or service into a market-ready offering that generates revenue while considering ethics, equity, and access.
- Social Innovation—A new idea, model, or service in health that addresses unmet needs, improves outcomes, and often involves collaboration across sectors.
- Sustainability—The ability of a health program or model to maintain its impact over time through stable funding, stakeholder support, and integration into routine practice.

## Data Availability

The original contributions presented in the study are included in the article/Supplementary Material, further inquiries can be directed to the corresponding author.

## Footnotes

1 About Learning Health Systems. Content last reviewed May 2019. Agency for Healthcare Research and Quality, Rockville, MD. [Available from: https://www.ahrq.gov/learning-health-systems/about.html].

2 NICU Environment. Content last reviewed 2024. FiCare Family Integrated Care, Sinai Health System, Toronto, Canada. [Available from: https://familyintegratedcare.com/the-ficare-approach/program-development/nicu-environment/].

3 Family Integrated Solutions Calgary, Canada: 2025 *Liminality Innovations Inc;* 2025 [Available from: https://liminality.ca/].
