# Correction: Social enterprise as a strategy to advance patient-oriented health services innovation: learning from the Alberta Family Integrated Care model

**DOI:** 10.3389/frhs.2025.1758571

**Published:** 2026-01-02

**Authors:** Anmol Shahid, Kristen Graham, Karen Benzies

**Affiliations:** 1 Faculty of Nursing, University of Calgary, Calgary, AB, Canada; 2 Faculty of Nursing, Alberta Health Services, Calgary, AB, Canada; 3 Faculty of Nursing, Liminality Innovations, Calgary, AB, Canada

**Keywords:** delivery of care, neonatal, intensive care units, learning health system, sustainability, social innovation, social enterprise, critical care

**Correction 1:**

In the published article, there was a mistake in the first sentence of Section 3.3.4 (paragraph 1). The sentence appeared to imply that many metrics for Liminality Inc. were unavailable although they are available for the research phases of the Alberta FICare work (called MERGE in the manuscript). The statement currently says [“not yet able to comprehensively report on metrics such as cost savings for health systems generated by our products, stakeholder retention, or the specifics of Alberta FICare uptake beyond Alberta.”]

The correct statement is [“*Liminality Innovations Inc.* is a young organization and has published research papers on Alberta FICare metrics such as health system utilization and cost avoidance for the health system and out of pocket costs for parents (24, 30, 31, 40).”]

The original version of this article has been updated.

**Correction 2:**

In the published article, the governance structure was unlcear in sentence three of Section 3.3.4 (paragraph 1). The section currently states that [“However, we can report that currently, our CEO does not receive a salary, and the governance structure is composed largely of academic researchers who do not receive revenue from the company.”]

The correct statement is [“However, we can report the governance structure is comprised of a mix of academic researchers and business executives.”]

The original version of this article has been updated.

**Correction 3:**

In the published article, we have offered some information about the CEO's salary in sentence three of Section 3.3.4 (paragraph 1). While this is correct, we would like to move this statement to the conflict of interest statement for better alignment. The section stated that [“However, we can report that currently, our CEO does not receive a salary, and the governance structure is composed largely of academic researchers who do not receive revenue from the company.”]

The correct statement is [“Currently, our CEO does not receive a salary.”]. This statement should be placed in the “Conflict of interest” section, just after the sentence “Karen Benzies is the founder and Chief Executive Officer of *Liminality Innovations Inc.*, the social enterprise highlighted in this community case study.”

The original version of this article has been updated.

